# A mosquito salivary gland protein partially inhibits *Plasmodium* sporozoite cell traversal and transmission

**DOI:** 10.1038/s41467-018-05374-3

**Published:** 2018-07-25

**Authors:** Tyler R. Schleicher, Jing Yang, Marianna Freudzon, Alison Rembisz, Samuel Craft, Madeleine Hamilton, Morven Graham, Godfree Mlambo, Abhai K. Tripathi, Yue Li, Peter Cresswell, Photini Sinnis, George Dimopoulos, Erol Fikrig

**Affiliations:** 1 Section of Infectious Diseases, Department of Internal Medicine, Yale University School of Medicine, New Haven, Connecticut 06520 USA; 2 Department of Dermatology, Yale University School of Medicine, New Haven, Connecticut 06520 USA; 3 Yale Center for Cellular and Molecular Imaging, Yale University School of Medicine, New Haven, Connecticut 06510 USA; 4 Department of Molecular Microbiology and Immunology, Bloomberg School of Public Health, Johns Hopkins University, Baltimore, Maryland 21205 USA; 5 Department of Immunobiology, Yale University School of Medicine, New Haven, Connecticut 06520 USA; 6 Howard Hughes Medical Institute, Chevy Chase, Maryland 20815 USA

## Abstract

The key step during the initiation of malaria is for motile *Plasmodium* parasites to exit the host dermis and infect the liver. During transmission, the parasites in the form of sporozoites, are injected together with mosquito saliva into the skin. However, the contribution of vector saliva to sporozoite activity during the establishment of the initial infection of the liver is poorly understood. Here we identify a vector protein by mass spectrometry, with similarity to the human gamma interferon inducible thiol reductase (GILT), that is associated with saliva sporozoites of infected *Anopheles* mosquitoes and has a negative impact on the speed and cell traversal activity of *Plasmodium*. This protein, referred to as mosquito GILT (mosGILT) represents an example of a protein found in mosquito saliva that may negatively influence sporozoite movement in the host and could lead to new approaches to prevent malaria.

## Abstract (web summary)

Mosquito saliva can affect transmission of *Plasmodium* to mammalian hosts, but active saliva components or mechanisms are poorly understood. Here, the authors identify a mosquito saliva protein that binds *Plasmodium* sporozoites and inhibits cell traversal in vitro and sporozoite speed in mice.

## Introduction

Malaria remains one of the deadliest diseases worldwide^1^. The key step during the initiation of malaria is for the *Plasmodium* parasite to infect the host liver. After insertion of the proboscis from an infected female *Anopheles* mosquito, *Plasmodium* parasites, in the form of sporozoites, are injected together with mosquito saliva into the skin of an animal host. Sporozoites within the dermis must migrate to a blood vessel that will transport them to the liver. Once at the liver, sporozoites invade hepatic cells and develop into exoerythrocytic forms (EEFs) containing thousands of merozoites, which are released into the circulation and establish a blood-stage infection^1–4^.

Motility is essential for *Plasmodium* to properly navigate through both the host skin and hepatic microenvironments. Sporozoites utilize substrate-dependent gliding motility and cell traversal in both locations as they travel toward the host hepatocytes^5–13^. Within the skin, sporozoites are able to switch from an initial rapid motility to a more restricted movement around the dermal blood vessels^7^. In addition, as sporozoites move towards the liver, they can enter and exit host cells within transient vacuoles, which are molecularly distinct from the parasitophorous vacuole membrane used during a productive hepatocyte infection^14^. This process, known as cell traversal, allows the sporozoites to cross cellular barriers and evade the host immune response^5,8,13,15,16^. Numerous sporozoite proteins involved in cell traversal and gliding motility have been identified^6,8,14,15,17–22^. However, many additional signals and environmental factors regulating sporozoite motility in the host still remained to be determined.

Mosquito saliva contains numerous proteins that work as efficient immunomodulators, in addition to molecules with anti-hemostatic and vasodilatory functions^23,24^. The main purpose of these saliva components is to facilitate blood feeding, but they could also have an influence on pathogen transmission. Saliva proteins can have an impact on a microorganism indirectly, by altering the microenvironment and enhancing transmission^24–26^. As one of several examples, maxadilan, a vasodilator and immunomodulator in sandfly saliva, exacerbates *Leishmania major* infection^26–30^. Other proteins in vector saliva can directly interact with pathogens and influence transmission. As one example, the tick saliva protein, Salp15, binds the surface of *Borrelia burgdorferi* and inhibits complement-mediated killing^31,32^. Studies have examined proteins in mosquito salivary glands (SGs) that are important for the survival of *Plasmodium* within the vector, such as saglin^33^. In addition, a recent study described that immunization against an abundant *Anopheles* specific SG protein, AgTRIO, can reduce the parasite burden in the host after mosquito-borne transmission^34^. However, investigators have not characterized proteins in mosquito saliva that directly interact with *Plasmodium* during movement out of the vector. Therefore, we examined *Plasmodium* sporozoites purified from mosquito saliva in an effort to identify targets that may be manipulated to interfere with the malaria transmission. Here we show a mosquito SG protein with homology to the human gamma interferon inducible thiol reductase (GILT) that interacts with the surface of *Plasmodium* sporozoites as they are expelled from *Anopheles* mosquito SGs. We found that this mosquito GILT-like protein can partially reduce the speed and cell traversal activity of both human and rodent *Plasmodium* sporozoites. The partial inhibition of these critical motility components modestly influences the ability of the sporozoites to migrate to the liver and establish a normal hepatic infection. This vector-parasite interaction may represent an example of how *Plasmodium *sporozoites optimize the number of parasites required to complete their life cycle or could suggest a lingering effect of the vector innate immune response that continues to limit parasite activity during transmission. Overall, characterizing the interaction between mosquito GILT and *Plasmodium* sporozoites could potentially help uncover new pathways associated with motility regulation of *Plasmodium* parasites and lead to the design of novel therapeutics to prevent malaria transmission.

## Results

### Mass spectrometry of sporozoites from vector saliva

**Fig. 1:**
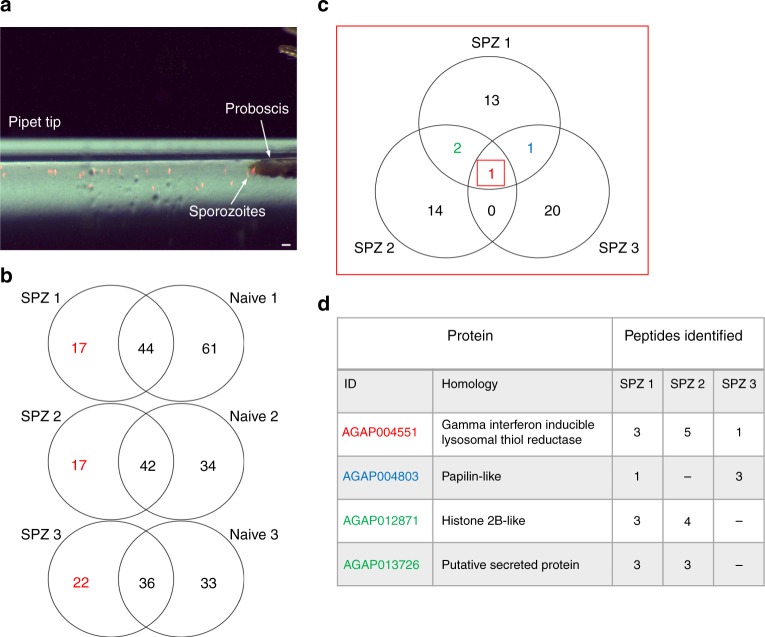
Mass spectrometry identified AGAP004551 in association with sporozoites purified from mosquito saliva. **a** An image depicting the collection of *Plasmodium* sporozoites from the saliva of infected mosquitoes in a pipet tip (sporozoites in red, scale 10 μm). **b** The mosquito proteins associated with sporozoites collected from saliva of infected mosquitoes (SPZ) were compared to residual mosquito proteins from naive saliva (naive) by LC MS-MS. Three independent biological replicates were used. Each Venn diagram represents the *A. gambiae* proteins associated with each fraction (*n* = 35–50 mosquitoes/sample). The numbers in red indicate proteins that were only present in the samples from *Plasmodium*-infected mosquitoes. **c** The *A. gambiae* proteins unique to each SPZ sample were then compared to each other to determine potential common proteins. **d** AGAP004551 was identified in all three experiments and several peptides were discerned in two of the experiments. The proteins identified in two of the independent experiments are highlighted in blue and green, respectively

To identify mosquito proteins in saliva that may interact with *Plasmodium*, sporozoites were collected directly from the saliva of infected *Anopheles gambiae* mosquitoes (Fig. 1a). Saliva from age-matched naive mosquitoes was collected in the same manner as a control. After washing the sporozoites, liquid chromatography tandem mass spectrometry (LC MS-MS) was utilized to identify potential mosquito proteins that might be strongly associated with the sporozoites in saliva. A complete list of all of the *A. gambiae* proteins identified with the saliva sporozoites, from three separate experiments, is provided in Supplementary Data 1. One mosquito protein, AGAP004551, was detected in all three independent biological replicates associated with *Plasmodium berghei* sporozoites (Fig. 1b, c). In two of the replicates, two or more AGAP004551 peptides were identified in each sample, and in one replicate AGAP004551 was identified by a single peptide (Fig. 1d). Three other mosquito proteins were also identified in two of the sporozoite replicates—a histone 2B-like protein, a protein with homology to papilin, and an unknown protein with a predicted signal peptide (Fig. 1d). In an additional study, to extend this observation to a *Plasmodium* species that infects humans, we utilized a colony of *Anopheles stephensi* mosquitoes infected with the human malaria parasite, *Plasmodium falciparum*. ASTE004050, the *A. stephensi* homolog to AGAP004551 (85% identical) was identified along with *P. falciparum* sporozoites collected from *A. stephensi* mosquitoes (Supplementary Data 1).

### AGAP004551 is expressed in *Anopheles gambiae* SGs

**Fig. 2:**
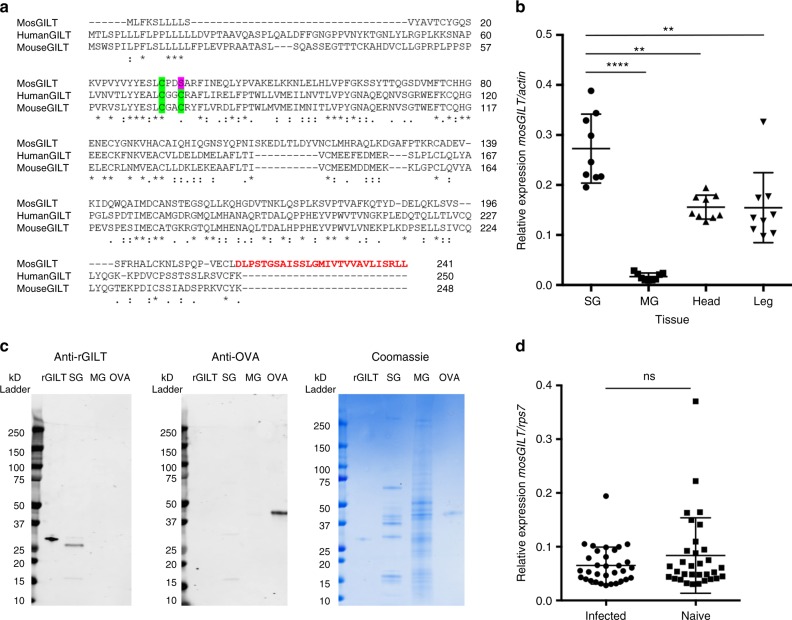
AGAP004551 (mosGILT) is a gamma interferon inducible lysosomal thiol reductase-like protein expressed in mosquito salivary glands. **a** An alignment of the *A. gambiae* AGAP004551 (mosGILT) sequence with the human GILT and mouse GILT sequences (Key * -conserved residues, : -strongly similar residues, . -weakly similar residues). The active site cysteines of mosquito, human, and mouse GILT are highlighted in green and the mosGILT active site serine in pink. The C-terminus of mosGILT is highlighted in bold red. **b** RT-qPCR of mosquito tissues (data pooled from three independent experiments, each point represents three mosquito tissues, mean ± standard deviation (SD), *n* = 9/group, unpaired *t*-test, **p < 0.005, ****p < 0.00001). **c** Western blots and SDS-PAGE of mosquito salivary glands (SG) and midguts (MG). Western blots were probed with polyclonal mouse anti-rGILT (recombinant mosGILT) or mouse anti-Ovalbumin sera (OVA) as a control at 1:1000 dilutions (kilodaltons, kD). **d** RT-qPCR of *P. berghei* infected and naive salivary glands. Each point represents an individual mosquito salivary gland (data pooled from four independent experiments, *n* = 32/group, mean ± SD, ns not significant)

*A. gambiae* AGAP004551 has homology to the human GILT and is now named mosGILT. MosGILT is 19% and 19.5% identical to the human and mouse GILT proteins, respectively. The human and mouse GILT active sites are characterized by two reactive cysteines (CxxC) that reduce disulfide bonds^35,36^. In contrast, mosGILT has a serine in the second cysteine position (Fig. 2a). Another major difference between the human/mouse GILT and mosGILT sequences is an extended C-terminus composed of hydrophobic amino acid residues (Fig. 2a). Purified recombinant mosGILT (rGILT) did not show any detectable thiol reducing activity at pH 4.5 or pH 7.4 compared to human GILT (CxxC active site) and mouse GILT C2 (CxxS active site; Supplementary Fig. 1). The mutated mouse GILT C2 still had reducing activity, but lower than the native CxxC human GILT (Supplementary Fig. 1).

Transcripts of *mosGILT* were detected in multiple tissues (Fig. 2b). Female SGs expressed significantly higher levels of *mosGILT* compared to midguts and the other tissues analyzed (Fig. 2b, *p* < 0.00001, unpaired *t*-test). Antibodies to rGILT recognized endogenous mosGILT in the SGs by western blot (Fig. 2c). The predicted molecular weight of endogenous SG mosGILT is 26.8 kD and is similar to the observed mass seen by western blot. Sera from mice immunized with ovalbumin (OVA) was used as a control to demonstrate the specificity of sera from mice immunized with rGILT. In addition, anti-rGILT also confirmed the limited mosGILT protein content of the mosquito midgut (MG) compared to the SG, as seen in previous mRNA analyses (Fig. 2b, c). Infection of the SGs by *Plasmodium* did not alter the expression of *mosGILT* (Fig. 2d). While mosGILT is an abundant protein in *A. gambiae* SGs, it does not appear to be present at detectable levels as a soluble component of saliva (Supplementary Fig. 2a). This suggests that the interaction between mosGILT and *Plasmodium* potentially begins at some point in the SGs and then allows the protein to travel in association with the sporozoites into the mammalian host. Mass spectrometry of *A. gambiae* SG extracts revealed that mosGILT is one of the top 100 detected proteins based on spectral count comparisons, a good indicator of relative abundance (#76 from over 850 proteins identified; Supplementary Data 2). By comparing the relative intensities of immunoblot bands from known concentrations of rGILT, the endogenous levels of mosGILT in vector SGs was estimated to be 15 ng/SG pair (Supplementary Fig. 3a, b; the total protein content of an adult *Anopheles* SG is around 1 µg). The total volume of mosquito SGs is low and can be estimated to be around 5 nanoliters (assuming each lobe is similar to a cylinder), allowing sporozoites to be in the presence of high concentrations (112 µM) of mosGILT protein within the vector (Supplementary Fig. 3c, d). Furthermore, using a similar approach, the amount of mosGILT associated with sporozoites per mosquito was estimated to be 0.12 ng/mosquito or 4.5 µM if assuming the mosquito produced 1 nanoliter of saliva (Supplementary Fig. 2a, b).

### MosGILT binds to the *Plasmodium berghei* sporozoite surface

**Fig. 3:**
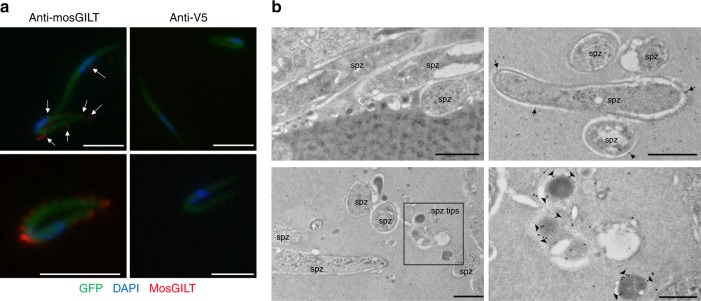
MosGILT binds to the surface of *P. berghei* sporozoites. **a** Immunostaining with a mosGILT specific monoclonal antibody revealed endogenous SG mosGILT bound to the surface of *P. berghei* sporozoites. An irrelevant mouse monoclonal, anti-V5, was used as a control. All images are representative of more than three independent experiments. MosGILT bound to sporozoites is highlighted by white arrows (Scale - 5 μm). **b** Immunoelectron microscopy labeling of endogenous mosGILT on *P. berghei* sporozoites within intact SGs. Sporozoites labeled with a control mouse serum (top left). Sporozoites labeled with the anti-mosGILT monoclonal antibody, top right and bottom panels. The region highlighted by a black square represents a higher magnification of predicted sporozoite anterior or posterior tips, bottom right. Arrows highlight mosGILT labeling at the surface of sporozoites (spz-sporozoite; scale 1 μm and 500 nm bottom right)

To further examine the mosGILT interaction with *Plasmodium* sporozoites, a monoclonal antibody specific to mosGILT was used to immunostain *P. berghei* sporozoites isolated from infected SGs (Fig. 3a). Sporozoites had detectable staining of endogenous mosGILT at the cell surface. Some sporozoites isolated directly from the SGs appeared to have an enrichment of mosGILT staining on the surface. Notably, sporozoites with elevated levels of mosGILT staining were often detected in a folded morphology (Fig. 3a). In addition, sporozoites from intact SGs were labeled with anti-mosGILT and imaged using immunoelectron microscopy (Fig. 3b). Within the SG microenvironment, *P. berghei* sporozoites also contained surface labeling of mosGILT. In some images, mosGILT labeling was prominent near the tips of the sporozoites (Fig. 3b). Together, these microscopy studies reveal that mosGILT interacts with the surface of SG sporozoites within their natural microenvironment.

### *Plasmodium* cell traversal activity is reduced by rGILT

**Fig. 4:**
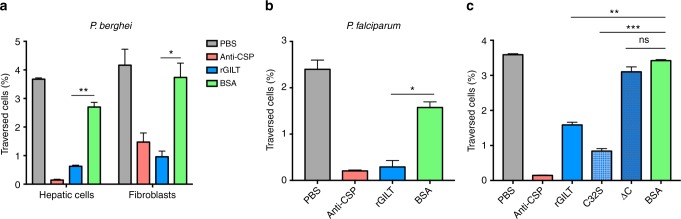
Sporozoite cell traversal is inhibited by rGILT. Cell traversal assay of **a** *P. berghei* sporozoites preincubated with rGILT (80 µg/ml- 3 µM) and then incubated with murine hepatic cells or murine dermal fibroblasts. **b** Cell traversal assay of *P. falciparum* 3D7 sporozoites incubated with rGILT (20 µg/ml- 0.7 µM) and then incubated with human hepatic cells. Cell traversal by sporozoites was analyzed on a Stratedigm flow cytometer by measuring the percentage of fluorescent dextran positive cells. **c** Cell traversal assay of murine hepatic cells and *P. berghei* sporozoites preincubated with either rGILT, rGILT∆C, rGILT C32S, BSA (all 80 µg/ml), or anti-CSP. Bovine serum albumin (BSA; 80 µg/ml), a protein with no effect on sporozoite traversal, and the monoclonal antibodies to the *P. berghei* or *P. falciparum* circumsporozoite protein (CSP, 3D11 or 2A10, respectively, 300 µg/ml), known inhibitors of traversal, were used as controls. Data are representative of more than three experiments (mean ± SD, unpaired *t*-test, *p < 0.05, **p < 0.005, ***p < 0.0005, ns not significant)

Aside from within the vector SG, the interaction between sporozoites and mosGILT occurs in the host skin, after inoculation by the mosquito and prior to sporozoite invasion of the host liver. Therefore, we assessed the ability of mosGILT to influence cell traversal, an important strategy used by sporozoites as they migrate toward a final hepatocyte^5,37,38^. *P. berghei* sporozoites incubated with rGILT traversed a significantly lower number of hepatic cells (*p* < 0.005, unpaired *t*-test) and dermal fibroblasts (*p* < 0.05, unpaired *t*-test) compared to sporozoites incubated with bovine serum albumin (BSA) or an additional control that lacked a protein component (Fig. 4a). *P. falciparum* sporozoites incubated with rGILT also demonstrated a lower traversal efficiency compared to sporozoites incubated with control proteins (*p* < 0.05, unpaired *t*-test), demonstrating that this phenotype extends to a human pathogen (Fig. 4b). As an additional control, skin fibroblasts and hepatic cells were preincubated with rGILT or BSA prior to the traversal assay to determine if rGILT interacts with the cells in culture to create an indirect inhibition of sporozoite traversal. Pretreatment of the cell cultures with rGILT did not show any inhibition of sporozoite traversal, demonstrating that rGILT directly influences the traversal activity of the sporozoites (Supplementary Fig. 4). MosGILT has notable amino acid differences from human and mouse GILT protein sequences, which were targeted to generate different mosGILT protein constructs (Fig. 2a) and then tested using the established cell traversal activity assay. Deletion of the final 27 amino acids from the C-terminus of rGILT (rGILT ∆C) completely abolished the inhibitory activity of rGILT on cell traversal (Fig. 4c). In addition, mutation of the first and only cysteine in the mosGILT predicted active site to a serine (rGILT C32S) did not alter the ability of the protein to inhibit cell traversal (Fig. 4c). The concentration of rGILT used in these in vitro studies (80 µg/ml; 3 µM) is similar to the predicted concentration of endogenous mosGILT found in saliva with sporozoites (~4.5 µM; Supplementary Fig. 2).

*Plasmodium* sporozoites treated with rGILT have a similar viability compared to those treated with PBS or BSA (Supplementary Fig. 5a). Only the anti-circumsporozoite protein (CSP) monoclonal antibody, known to immobilize sporozoites in vitro^12,39^, reduced the survival of *Plasmodium* (*p* < 0.05, unpaired *t*-test), indicating that the presence of rGILT does not inhibit cell traversal by a direct toxic effect on the parasite (Supplementary Fig. 5a). In addition, sporozoites treated with rGILT developed a significantly higher percentage of EEF-positive cells compared to control treated parasites, as measured by flow cytometry 48 h after infection in vitro (*p* < 0.05, unpaired *t*-test, Supplementary Fig. 5b). Regulation of traversal is essential for determining the outcome of a productive infection. Previous studies of sporozoites deficient in traversal also noted the ability of the parasites to develop EEFs at a higher rate than wild-type sporozoites^5,11,14^. The increase in EEF development in vitro also supports the finding that rGILT does not alter the viability of sporozoites.

**Fig. 5:**
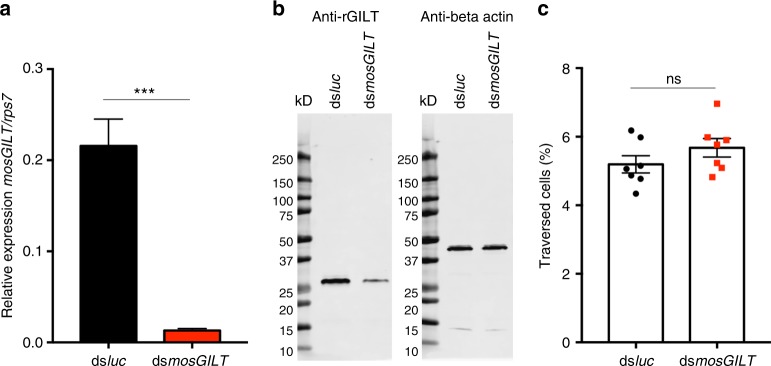
Analysis of a partial knock-down of *mosGILT* and the impact on sporozoite cell traversal (**a**) RT-qPCR of SGs injected with either dsRNA targeting an irrelevant *luciferase* gene or *mosGILT*. Data represent duplicate experiments from more than three experiments (mean ± SD, unpaired *t*-test, ***p < 0.0005). **b** Western blots of SGs from ds*luc* or ds*mosGILT* injected mosquitoes. **c** Cell traversal assay of murine hepatic cells incubated with *P. berghei* sporozoites isolated from ds*luc* or ds*mosGILT* salivary glands (2000 sporozoites/well). Cell traversal by sporozoites was analyzed on a Stratedigm flow cytometer by measuring the percentage of fluorescent dextran positive cells. Each point represents a technical replicate pooled from two independent experiments (*n* = 7, ns not significant)

To study the interaction of endogenous mosGILT on sporozoite activity, double stranded (ds) *mosGILT* RNA was injected into *P. berghei*-infected mosquitoes. Silencing SG genes in mosquitoes has been well known to be less efficient than genes targeted in other mosquito tissues and requires the injection of high concentrations of dsRNA^40^. Seven days after injection of the dsRNA, expression of *mosGILT* was reduced greater than 90% compared to a dsRNA injected control group (Fig. 5a; ds*luciferase*; ds*luc*). Analysis of mosGILT protein levels in the SGs by western blot, also revealed a reduction, but not a complete elimination of mosGILT protein (Fig. 5b). In order to determine if a reduction in mosGILT has an impact on sporozoite cell traversal, sporozoites were harvested from dsRNA injected SGs and incubated with hepatocytes (Fig. 5c). Only a modest increase in cell traversal, that did not reach statistical significance, was detected between the experimental and control sporozoites, likely because the reduction in mosGILT was not complete (Fig. 5b, c). Generating a mosquito lacking *mosGILT* using the CRISPR/Cas9 approach^41,42^ will be an alternative to further explore the role of mosGILT in sporozoite cell traversal and transmission.

### Infection of the murine liver is partially reduced by rGILT

**Fig. 6:**
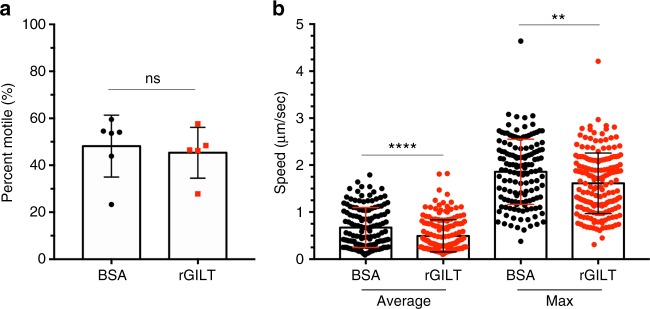
Recombinant mosGILT limits the speed of sporozoites in vivo. Sporozoites were analyzed by two-photon microscopy after incubation with rGILT or BSA (80 µg/ml). **a** Percentage of motile and non-motile sporozoites detected in murine skin after intradermal injection (*n* = 6 BSA mice, *n* = 316 total sporozoites; *n* = 5 rGILT mice, *n* = 412 total sporozoites). **b** Average speed and max speed of motile sporozoites for tracks that displaced greater than 15 μm (*n* = 6 BSA mice, *n* = 140 total sporozoites; *n* = 5 rGILT mice, *n* = 178 total sporozoites) Data represent three independent experiments (mean ± SD, unpaired *t*-test, **p < 0.005, ****p < 0.0001, ns not significant)

As rGILT has inhibitory activity on cell traversal, a behavior important for sporozoites to infect a host, we tested whether rGILT had an impact on sporozoite infectivity in vivo. Sporozoite motility was analyzed after intradermal injection into murine ears using two-photon microscopy, a powerful technique, in addition to several other in vivo imaging approaches, that have been used to observe sporozoite movement within host tissue^7,12,34,43^. There was no difference in the percentage of motile and non-motile parasites between BSA and rGILT incubated sporozoites (Fig. 6a). Analysis of only the motile sporozoites in both groups revealed that rGILT-treated sporozoites were significantly slower than BSA-treated sporozoites when measuring the average and max speed over individual tracks that displaced at least 15 µm within the murine dermal tissue (Fig. 6b, Average *p* < 0.0001 and Max *p* < 0.005, unpaired *t*-test).

**Fig. 7:**
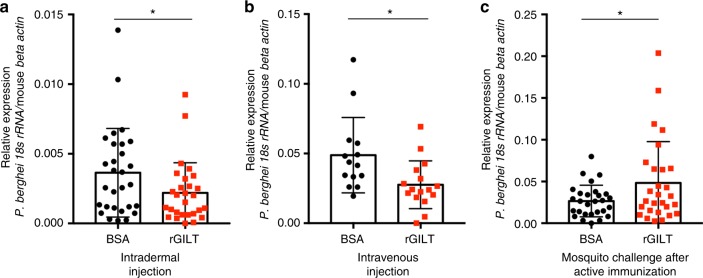
MosGILT negatively impacts the transmission of *P. berghei* sporozoites. **a** Liver *Plasmodium* burden 40 hours after intradermal injection of 2500 *P. berghei* sporozoites incubated with either rGILT or BSA (100 µg/ml each). Data pooled from five independent experiments (mean ± SD, *n* = 29 BSA, *n* = 28 rGILT, Welch’s *t*-test, *p < 0.05). **b** Liver *Plasmodium* burden 40 hours after intravenous injection of 2000 rGILT or BSA-treated sporozoites (100 µg/ml each). Data are pooled from two independent experiments (mean ± SD, *n* = 14 BSA, *n* = 16 rGILT, Welch’s *t*-test, *p < 0.05). **c** Mice actively immunized with either rGILT or ovalbumin (OVA) were each bitten by three *P.* *berghei*-infected *A. gambiae* mosquitoes. The liver *Plasmodium* burden was analyzed 40 hours after the infectious bites. Data are pooled from three independent experiments (mean ± SD, *n* = 29 Ova, *n* = 29 rGILT, Welch’s *t*-test, *p < 0.05)

Examination of murine livers by RT-qPCR 40 hours after intradermal or intravenous injection of *P. berghei* sporozoites revealed significant decreases in the level of liver infection by rGILT-treated sporozoites compared to BSA-treated control sporozoites (Fig. 7a, b, *p* < 0.05, Welch’s *t-*test). *P. berghei* sporozoites treated with rGILT (*n* = 28, mean 0.00219) displayed a 40% reduction in the liver burden compared to control sporozoites (*n* = 29, mean 0.00364) following intradermal injection (Fig. 7a). In addition, rGILT-treated sporozoites revealed a similar significant reduction in the liver burden after intravenous injection (Fig. 7b, 43% reduction, rGILT *n* = 16, mean 0.0276 and BSA *n* = 14, mean 0.0488, *p* < 0.05, Welch’s *t*-test). Analysis of the blood-stage of malaria, 3 to 8 days post infection (dpi), did not reveal any significant differences in the presence of blood-stage parasites originating from sporozoites treated with rGILT or BSA (*n* = 17/group; Supplementary Fig. 6). This indicates that after the parasite has had ample time to replicate in the host and left the liver for the bloodstream, the initial effects of rGILT on early infection are no longer apparent.

In order to demonstrate that mosGILT plays a role during biological transmission of *Plasmodium*, mice were actively immunized with rGILT or OVA and challenged with *P. berghei*-infected mosquitoes. Sera from OVA-immunized mice did not recognize rGILT (Supplementary Fig. 7a, b). In contrast to the lower liver burdens detected after injection with sporozoites treated with rGILT, mice immunized with rGILT and exposed to infectious mosquito bites developed significantly higher liver parasite burdens than OVA-immunized mice (Fig. 7c; *n* = 29 OVA, *n* = 29 rGILT, 82% increase, *p* < 0.05, Welch’s *t*-test). Mosquitoes used to infect both groups of mice carried similar levels of sporozoites (Supplementary Fig. 7c). Together these results further demonstrate that mosGILT can modulate the level of initial *Plasmodium* infection in mice.

## Discussion

The influence of vector saliva on the transmission of pathogenic microorganisms has been well documented for viral^44–46^, bacterial^31,32^, and parasitic diseases^25,47^. Overall, it is believed that saliva promotes transmission of various pathogens, either by altering the host microenvironment or by directly interacting with the microorganism^24,25^. Studies regarding the impact of mosquito saliva on *Plasmodium* transmission have been conflicting^48–50^. In addition, there are limited studies focusing on the influence of specific mosquito saliva proteins (positive or negative) on the transmission of *Plasmodium* sporozoites. Only recently a study discovered that immunization of mice with an *Anopheles* specific saliva protein, AgTRIO, can reduce the ability of *Plasmodium* to infect the host after mosquito-borne transmission^34^. In our study, we have identified a mosquito SG protein with homology to the mammalian GILT that associates with *Plasmodium* sporozoites as they are expelled from the vector salivary duct and reduces the ability of the parasite to infect the host. More importantly, we demonstrate that the interaction between this mosquito protein (mosGILT) and sporozoites also applies to *Plasmodium falciparum*, the pathogen responsible for the greatest number of fatal cases of human malaria^1^.

As mosGILT is normally a SG protein and not a component of *Anopheles* saliva, it is likely that this interaction between mosGILT and *Plasmodium* originates from within the SG microenvironment, where the possible function of mosGILT is to restrict or limit parasite activity inside the SG. Microscopy experiments analyzing the association of endogenous SG mosGILT and *Plasmodium* within the SG showed that mosGILT can bind to the surface of these sporozoites (Fig. 3). A previous study has described altered sporozoite motility within the SG and salivary duct compared to sporozoites in host tissue^43,51^. However, the impact of mosGILT on sporozoite activity within the vector SGs will require further characterization.

In the presence of rGILT, *P. berghei* sporozoites displayed a reduced ability to infect murine livers after needle inoculation (Fig. 7a, b). Although the presence of rGILT altered the initial infection of murine livers by sporozoites, there was no significant effect on the outcome of blood-stage parasitemia, suggesting that after pathogen replication, the infection is able to recover (Supplementary Fig. 6). Under natural biological transmission, mice immunized with rGILT provided evidence that rGILT-specific antibodies can neutralize the inhibitory activity of mosGILT on *Plasmodium* sporozoites after transmission from infected mosquitoes, allowing the establishment of larger hepatic infections (Fig. 7c). Antibodies specific to mosGILT could have a stronger affinity for this vector protein compared to the currently unknown *Plasmodium* binding partner, which could neutralize the mosGILT away from the surface of the sporozoites, thus allowing more sporozoites to migrate towards the liver. At this time, it is also unknown if there is a small portion of free mosGILT in the environment during transmission that can influence sporozoites. However, these data indirectly provide further evidence that mosGILT is expelled from the vector, along with sporozoites, into the mammalian host. Once deposited in the skin, *Plasmodium* sporozoites are motile and need to find a blood vessel in order to successfully locate the host liver^1,3,7,43^. Cell traversal is an essential strategy that allows sporozoites to cross cellular barriers and evade the host immune response as they advance towards infecting the host liver^8,11^. Importantly, we found that cell traversal was significantly inhibited by rGILT (Fig. 4). Moreover, two-photon microscopy revealed that rGILT sporozoites are significantly slower while moving through the murine dermis (Fig. 6). Previous research has also demonstrated that *Plasmodium* sporozoites deficient in cell traversal or motility display a reduced ability to infect vertebrate hosts, but can still infect hepatic cells in vitro or in mice treated with clodronate, a drug that removes Kupffer cells^8,15,17,18,21,22^. Although sporozoite cell traversal is robustly inhibited in vitro (Fig. 4), our in vivo infection studies in murine hosts revealed a much more modest effect on *Plasmodium* transmission (Fig. 7). Sporozoite motility is well known to be influenced by different physiological conditions, such as a 2D or 3D environment^10,52–54^. Once in vivo, sporozoites likely encounter additional attachments sites within the host tissue, which could help *Plasmodium* to overcome the impact of rGILT. Overall our results reveal that mosGILT, a mosquito protein, has a negative influence on sporozoite cell traversal and impairs the initial pathogenicity of *Plasmodium* in a vertebrate host.

The mosGILT amino acid sequence has several different features when compared to mammalian GILT proteins. In mammals, GILT functions as a thiol reductase which enables the efficient digestion of proteins for antigenic presentation and a proper immune response^35,36,55^. Enzymatic activity of GILT is carried out by two reactive cysteines found in the active site. MosGILT contains a CxxS active site (Fig. 2a). Mutating the second cysteine has been shown to decrease the ability of GILT to reduce disulfide bonds^36^. The mutant GILT protein is still able to initiate the thiol reduction reaction, but not complete it. MosGILT does not have the ability to partially reduce a disulfide-containing substrate (Supplementary Fig. 1). Mammalian GILT is synthesized in a precursor form, after which N- and C-terminal propeptides are cleaved resulting in the mature form of GILT^36^. The N- and C- termini of mosGILT are notably different, especially the C-terminus of mosGILT, which contains more hydrophobic residues than mammalian GILT (Fig. 2a). This study revealed that the C-terminus of mosGILT is required for the inhibitory activity on sporozoite cell traversal (Fig. 4c).

There are several possibilities for why a vector protein, such as mosGILT, may negatively influence pathogen transmission in the vertebrate host. It is well accepted that arthropod-borne parasites do not want to kill their host, as this can limit their ability to complete their life cycle. *Plasmodium* sporozoites must balance the magnitude of the disease optimal for subsequent transmission back to the vector. Potentially, mosGILT bound sporozoites are sacrificed in an effort to reduce the chance of over-infecting the host. This has been postulated for why the parasite is coated with an abundant immunogenic protein, CSP^56^. MosGILT could also provide a selective pressure to ensure that only a subpopulation of sporozoites migrate toward the host liver, which might provide an evolutionary advantage to *Plasmodium*. In addition, previous studies have noted a large population of sporozoites which do not leave the skin^43,57^. Inactive sporozoites in the host dermis will need to be examined in more detail to determine if there is any connection between the activity of mosGILT and the observation that some parasites do not exit the skin.

The overall paradigm in vector biology is that proteins in arthropod saliva have the ability to enhance the transmission of pathogens. However, our study demonstrates that a specific protein originating from the mosquito SG can have unfavorable effects on *Plasmodium* transmission. Therefore, studies that examine the global effect of saliva or salivary extracts on pathogen transmission are important, but may sometimes yield incomplete results as individual beneficial or detrimental effects of specific saliva components are not independently accounted for. Examination of individual components which have inhibitory effects on pathogen transmission, such as mosGILT, may help to further extend this paradigm. In addition, it is possible that therapeutics could be developed that naturally mimic or enhance the activity of mosGILT and more robustly inhibit *Plasmodium* motility in the skin, leading to new strategies to prevent malaria.

## Methods

### Ethics statement

All mice were housed by the Yale Animal Resource Center at Yale University and handled according to the NIH Guide for the Care and Use of Laboratory Animals. The experiments designed for these studies were approved by the Institutional Animal Care and Use Committee of Yale University under the protocol number 2017-07941. Human blood for *Plasmodium falciparum* cultures and mosquito infections was collected from a pool of pre-screened donors under an institutional review board approved protocol at Johns Hopkins University (Protocol NA00019050).

### Animals

*Anopheles gambiae* (4arr strain, MRA-121, MR4, ATCC; Keele strain, Johns Hopkins Malaria Research Institute) and *Anopheles stephensi* (Liston strain) mosquitoes were raised at 27 °C, 80% humidity, under a 12/12-hour light/dark cycle and maintained with 10% sucrose. Swiss Webster and C57BL/6 mice (5-6-week-old females) were purchased from Charles River Laboratories (Wilmington, MA).

### *Plasmodium berghei* infection

*P. berghei* (ANKA GFPcon 259cl2, MRA-865, ATCC) was maintained by serial passage in 6–8 weeks old female Swiss Webster mice. Five days after infection, murine parasitemia was monitored by fluorescent microscopy using air-dried blood smears. Mosquitoes were deprived of sucrose for 20 hours and then fed on anesthetized mice with parasitemia levels greater than 1% (3–5 mice/cage of 200 mosquitoes). The blood-fed mosquito cages were immediately transferred to a chamber set to 20 °C and covered with a damp paper towel. Three days after feeding, bottles containing a cotton wick soaked in 10% sucrose supplemented with 0.5% penicillin/streptomycin were provided to the remaining mosquitoes and subsequently changed every three days. Sixteen to eighteen days after infection, mosquitoes were anesthetized on ice and the SGs were screened under a fluorescent microscope to confirm the presence of a *P. berghei* infection.

### *Plasmodium falciparum* infection

For *P. falciparum* sporozoites, female *A. gambiae* (Keele strain) or *A. stephensi* (Liston strain) were fed through an artificial membrane on a blood culture containing either *P. falciparum* NF54 (BEI Resources, MRA-1000) or 3D7HT-GFP (BEI Resources, MRA-1029) gametocytes (mature gametocytes were adjusted to 0.3% in 50% human red blood cells (O+) containing 50% human serum (O+); please refer to the ethics statement) at the Malaria Research Institute at the Johns Hopkins Bloomberg School of Public Health as previously described^58–60^. *P. falciparum*-infected mosquitoes were wing-clipped at Johns Hopkins University and transferred to Yale University for sporozoite cell traversal studies.

### Saliva sporozoite collection

Saliva containing sporozoites was collected from infected *Anopheles* mosquitoes in a similar manner as previously described^34,61^. Female mosquitoes (17–21 dpi) were anesthetized on ice and screened for the presence of a SG infection using a fluorescent microscope. The legs of the infected mosquitoes were removed and the body of the mosquito was positioned onto double sided tape fixed to a glass slide using the wings as a guide. The proboscis of the mosquito was gently inserted into a low retention tip (VWR) containing 5 µl of PBS. Salivation was induced by the addition of approximately 1 μl of 50 mg/ml pilocarpine in 100% ethanol (Sigma Aldrich) to the abdomen of the mosquito. After 30 min, saliva sporozoite containing pipet tips were pooled from 30–50 mosquitoes. Sporozoites were purified from the saliva by centrifugation at maximum speed (18,407× *g*) at 4 °C for 15 min. Sporozoites were washed with 100 μl of PBS one to three times at maximum speed for 10 min and tended to reside in a film within the residual volume rather than a tight cell pellet. Saliva sporozoites were stored at −80 °C until further analysis. Saliva from uninfected mosquitoes was collected under the exact same conditions to assess the background of potential non-sporozoite saliva interactions (referred to as a naive pellet).

### Mass spectrometry

Samples were analyzed by the Mass Spectrometry and Proteomics Resource Facility of the W.M. Keck Foundation Biotechnology Resource Laboratory at Yale University. Sporozoites were digested with trypsin and analyzed on a QExactive-Plus (Thermo) or an Elite-Orbitrap mass spectrometer equipped with a Waters nanoAcquity ultra high-pressure liquid chromatography system. MS/MS spectra were searched against a combined *Plasmodium*-*Anopheles* protein database (*A. gambiae*- vectorbase.org v3.7 and *P. berghei* ANKA plasmodb.org v11 (19,932 total sequences) or *A. stephensi* SDA-500_ vectorbase.org v1.4 and *P. falciparum* 3D7 plasmodb.org v11(19,040 total sequences)) using the Mascot algorithm. Proteins were considered significant with two or more identified peptides and an expectation value less than 0.05. However, for comparisons, all proteins including tentative identifications (proteins with one peptide) were included in the analysis. Mosquito proteins identified from the saliva sporozoite sample were compared with a naive saliva pellet. Proteins unique to the sporozoite sample were considered as candidate proteins for further analysis.

### Cloning

The full-length sequence of mosGILT (AGAP004551) was amplified from *A. gambiae* 4arr SG cDNA sequences and cloned into the TOPO TA cloning vector (Thermo Fisher Scientific) according to the manufacturer’s protocol. MosGILT without the native signal peptide was then subcloned into a pMT/BiP/V5-His-A *Drosophila* Expression Vector (Thermo Fisher Scientific) using NcoI and NotI restriction sites. In addition, mosGILT lacking the final 27 amino acids of the protein sequence (rGILT∆C) or mosGILT C32S were also cloned into the pMT/BiP/V5-His-A *Drosophila* Expression Vector (regions highlighted in Fig. 2a). The primers utilized for these cloning procedures are listed in Supplementary Table 1. The vectors were sequenced at the Yale University W.M. Keck DNA Sequencing Facility to confirm that the mosGILT constructs were inserted in frame. The mosGILT construct containing pMT vectors, along with a selection vector pCOHygro (Thermo Fisher Scientific), were transfected into *Drosophila* S2 cells (CRL-1963, ATCC) using calcium phosphate according to the manufacturer’s protocol (Thermo Fisher Scientific). Individual stable cell lines expressing the *A. gambiae* mosGILT constructs were maintained at room temperature in Schneider’s medium containing 10% fetal bovine serum, 1% penicillin/streptomycin, and 300 μg/ml hygromycin-B (Thermo Fisher Scientific).

### Insect protein expression

For the purification of the various protein forms of rGILT (native, ∆C, or C32S), S2 cells expressing each rGILT construct were collected and transferred into 400 ml of serum-free Schneider’s medium containing only 1% penicillin/streptomycin and maintained in a spinner flask at room temperature. Following a 16–20-hour adjustment to the serum-free conditions, the S2 cell culture was induced using 500 μM copper (II) sulfate. Four to five days post-induction, the culture was centrifuged at 500× *g* for 30 min and the supernatant containing soluble rGILT was collected and filtered with a 0.2 μm bottle top filter. Tween-20 (0.05%) and beta-mercaptoethanol (5 mM) were added to the supernatant and filtered an additional time with a 0.4 μm filter to remove any precipitated material. rGILT was purified from the supernatant using a Ni-NTA agarose column (Qiagen) and eluted in 50 mM NaH_2_PO_4_, 500 mM NaCl, and 250 mM imidazole. Purified rGILT was concentrated with a 15 ml 3 kD Amicon filter (EMD Millipore) and washed three times with PBS. Protein concentrations were determined using a BCA assay (Thermo Fisher Scientific) with a BSA standard curve.

### Monoclonal antibody generation

Monoclonal antibodies specific to mosGILT were generated by GenScript (Piscataway, NJ) using purified rGILT∆C. Individual hybridoma clones were screened by western blotting and ELISAs with the recombinant antigen and endogenous SG extract to confirm specificity to the native *A. gambiae* mosGILT. The hybridoma clone 4C10G9 was selected after best recognizing mosGILT by western blot and ELISA and having a high titer in hybridoma supernatant for purification. 4C10G9 was maintained in DMEM with 10% FBS and 1% penicillin/streptomycin at 37 °C and 5% CO_2_. For monoclonal antibody purification, cells were pooled, washed two times in PBS, and transferred into serum-free hybridoma medium (Thermo Fisher Scientific) containing 1% penicillin/streptomycin for 5 days at 37 °C and 5% CO_2_. The cells were removed from the supernatant by centrifugation at 200× *g* for 10 min. The supernatant was diluted 1:1 with Protein A/G binding buffer (Thermo Fisher Scientific). 4C10G9 antibody was purified using protein A/G agarose (Thermo Fisher Scientific) and concentrated in PBS with a 10 kD Amicon filter.

### SG sporozoite isolation

Sporozoites were released from the SGs by repeated passaging through a 28½ gauge insulin syringe (10 times). The sporozoite SG mixture was then filtered through a 40 μm sterile filter and centrifuged at 17,200× *g* for 10 min. The resulting sporozoite pellet was resuspended in 50–100 μl of PBS and counted using a hemocytometer. For immunostaining, the sporozoites were gently centrifuged onto the surface of 8-well chamber glass slides (Lab Tek II, Nunc) at 300× *g* for 5 min. The sporozoites were then fixed with 4% PFA for 1 h at room temperature and washed three times with 400 μl of PBS for 10 min each.

### Immunostaining

Sporozoites were blocked with 1% BSA in PBS for 30 min at room temperature and then incubated with either a mosGILT specific monoclonal (4C10G9; 1:100) or a mouse anti-V5 monoclonal antibody (Thermo Fisher Scientific, R960-25; 1:100) overnight at 4 °C. The slides were washed three times for 5 min with PBS and then incubated with a goat anti-mouse Alexa fluor 555 secondary antibody (Thermo Fisher Scientific, A-21422; 1:500) for 1 h. The slides were again washed three times for 5 min with PBS, mounted with Prolong Gold Anti-fade containing DAPI (Thermo Fisher Scientific), and sealed with nail polish. Sporozoites were viewed using an EVOS FL Auto Cell Imaging System (Thermo Fisher Scientific) and images were processed using FIJI (v. 2.0.0-rc-59/1.51k).

### Gene expression

Total RNA was extracted for mosquito tissues dissected in RLT buffer with a RNeasy Mini Kit (Qiagen). cDNA was prepared with an iScript kit (Bio-Rad) following the manufacturer’s protocol. Quantitative PCR was performed using iQ SYBR Green Supermix (Bio-Rad) on a CFX96 real time system (Bio-Rad). PCR involved an initial denaturation at 95 °C for 3 min, 40 cycles of 10 s at 95 °C, 10 sec at 60 °C, and 10 sec at 72 °C. Fluorescence readings were taken at 72 °C after each cycle. At the end of each reaction, a melting curve (65–95 °C) was analyzed to confirm the identity of the PCR product. Relative expression of *mosGILT* was normalized to *A. gambiae ribosomal protein S7* or *A. gambiae actin* mRNA (Supplementary Table 1) using the comparative Ct method^62^.

### Western blotting

Proteins were separated by SDS-PAGE using 4–20% Mini-Protean TGX gels (Bio-Rad) at 300 V for 20 min. Proteins were transferred onto a 0.2 μm nitrocellulose membrane for 30 min at 100 V in a Tris-Glycine transfer buffer with 25% methanol. Blots were blocked in 5% non-fat milk in PBS with 0.1% Tween-20 for 30 min. Primary antibodies were diluted in block and incubated with the blots for 1 h at room temperature or 4 °C overnight (mouse anti-rGILT sera 1:1000, mouse anti-OVA sera 1:1000, 4C10G9 mouse anti-mosGILT monoclonal 1:1000, mouse anti-beta actin monoclonal, Abcam 8224, 1:1000). Infrared fluorescent secondary antibodies, goat anti-mouse IRDye 680LT (LI^-^COR, 926-68020) were diluted in block at 1:5000 and incubated for 1 h at room temperature. The blots were washed in PBS with 0.1% Tween-20 three times for 5 min in between incubations and imaging. Following the final wash, the immunoblots were dried and imaged with a LI-COR Odyssey imaging system.

### Bacterial protein expression

MosGILT without the native signal peptide was subcloned into a pGEX-6-P2 vector (GE Healthcare) using BamHI and NotI restriction sites. The vector was sequenced at the Yale University W.M. Keck DNA Sequencing Facility to confirm that the mosGILT insert was in frame. The mosGILT-pGEX plasmid was transformed into BL21 chemically competent cells (Thermo Fisher Scientific). GST-mosGILT fusion protein expression was induced with 0.1 mM IPTG at 30 °C for 4 h. The cells were sonicated in PBS with complete EDTA-free Protease Inhibitors tablets (Roche). Soluble GST-mosGILT was purified from the supernatant using glutathione sepharose 4B following the manufacturer’s protocol (GE Healthcare). Purified GST-mosGILT was concentrated with a 15 ml 3 kD Amicon filter (EMD Millipore) and washed three times with PBS. Protein concentrations were determined using a BCA assay (Thermo Fisher Scientific) with a BSA standard curve.

### Traversal and productive invasion assays

Mouse hepatocytes (Hepa1-6, CRL-1830, ATCC) and dermal fibroblasts (Clone III8C, CRL-2017, ATCC) were cultured (3 × 10^4^/well; 100 μl in 96-well plates) in DMEM (L-Glutamine, 10% FBS, 100 U/ml penicillin/streptomycin; Life technologies) or McCoy’s 5 A modified medium (10% FBS, 100 U/ml penicillin/streptomycin, Thermo Fisher Scientific) for Hepa1-6 or IIIC8, respectively, at 37 °C; 5% CO^2^ for 1 day. For cell traversal, *P. berghei* ANKA sporozoites (17–24 dpi, 2.5 × 10^3^/well) were preincubated with rGILT protein (0–100 μg/ml in 20 μl; 60 min; 4 °C) or BSA as a control (100 μg/ml in 20 μl; 60 min; 4 °C). Sporozoites were transferred onto Hepa1-6 or III8C cells. Cocultures were incubated (120 min; 37 °C; 5% CO^2^) with fluorescently labeled dextran (10,000 MW; 0.2 mg/ml; Molecular Probes), washed twice with PBS, and trypsinized (25 μl; 0.05% Trypsin-EDTA, phenol red; GIBCO). The percentage of traversed cells (Dextran-Red^+^) was determined by flow cytometry (Stratedigm). Human hepatocyte HC-04 cells (BEI Resources, MRA-975) were cultured (3 × 10^4^/well; 100 μl in 96-well plates treated with ECL cell attachment matrix (EMD Millipore) in DMEM (same as above, Thermo Fisher Scientific). *P. falciparum* sporozoites (NF54 and 3D7-GFP) were used for HC-04 cell traversal as described above.

To measure EEF development, *P. berghei* ANKA sporozoites (17–24 dpi, 5.0 × 10^3^/well) were preincubated with rGILT protein (0–100 μg/ml in 20 μl; 60 min; 4 °C) or BSA as a control (100 μg/ml in 20 μl; 60 min; 4 °C). Sporozoites were transferred onto Hepa1-6 cells. Cocultures were incubated (48 h; 37 °C; 5% CO^2^), washed twice with PBS, and trypsinized (25 μl; 0.05% Trypsin-EDTA, phenol red; GIBCO). The percentage of EEF-positive cells (GFP) was determined by flow cytometry (Stratedigm).

### Active immunization

C57BL/6 mice (6-week-old females, Charles River) were immunized once with either 10 µg of rGILT or OVA emulsified in Complete Freud’s adjuvant (CFA; Thermo Fisher Scientific) followed by two boosts of the respective antigen within Incomplete Freud’s adjuvant (IFA; Thermo Fisher Scientific) every 14 days. Seven days after the final boost, 25 µl of serum was collected from each mouse and the titers were tested by an ELISA to confirm antigen specific antibodies for each individual mouse.

### Enzyme-linked immunosorbent assays

Microtiter plates (MaxiSorp 96-well plates, Thermo Fisher Scientific) were coated with 3 µg/ml ovalbumin, rGILT, or GST-GILT in PBS at 4 °C overnight. The following day unbound protein was removed and the wells were rinsed twice with PBS-T (PBS 0.1% Tween). The wells were blocked with 5% non-fat milk in PBS-T for 1 h at room temperature. Serum samples from individual mice were serially diluted in 100 µl of blocking buffer starting at 1:50 and incubated for 2 h at room temperature. After the primary antibody incubation, a goat anti-mouse HRP secondary antibody (Thermo Fisher Scientific, 31430) was added to the wells at a 1:5000 dilution for 1 h at room temperature. The wells were rinsed three times with 400 µl of PBS-T in between all incubations. 100 µl of SureBlue 1-component TMB substrate (SeraCare) was added to the wells for 30 min in the dark, followed by 100 µl of the stop solution. The absorbance was recorded for all wells at 450 nm using a spectrophotometer.

### Infection studies

Sporozoites were released from infected SGs as described above and counted with a hemocytometer. Sporozoites were then incubated with rGILT or BSA (100 μg/ml) for 1 h on ice. Two thousand sporozoites in 10 µl of PBS were administered intravenously by retro-orbital injection with a 31-gauge 0.3 cc insulin syringe (Easy-Touch) For intradermal injections, 2500 sporozoites in a volume of 10 µl were injected into the dermis of the murine ear (6–8-week-old female C57/BL6, Charles River) with a 31-gauge 0.3 cc insulin syringe (Easy-Touch). After the intradermal injection, a noticeable wheal was present in the dermis, indicating a successful injection.

For mosquito transmission studies, one day before challenging actively immunized mice (OVA or rGILT immunized 12-week-old female C57BL/6) with *P. berghei*-infected *A. gambiae*, mosquitoes were screened for infection under a fluorescent dissection microscope (Zeiss). Mosquitoes were separated into two infection categories, high and low, based on the GFP intensity visualized in the SGs. A mosquito was determined to be heavily infected when both SGs were brightly positive for GFP while a lightly infected mosquito contained one GFP positive SG of the pair. Three mosquitoes (2 random mosquitoes from the high group and one from the low group) were separated into disposable cups covered by a mesh netting and a damp paper towel. The following day, mice were anesthetized with 150 µl of 10% ketamine and 2% xylazine in PBS and challenged with the *P. berghei*-infected *A. gambiae* mosquitoes for 20 min in the dark. Feeding was confirmed by the presence of a blood meal in the gut. There were no detectable differences in the ability of the mosquitoes to obtain a blood meal between the rGILT and OVA-immunized mice.

Unfed mosquitoes were recorded and discarded. Mosquitoes with a blood-meal visible in the midgut were pooled corresponding to the mouse they were exposed to and frozen at −80 °C for comparing approximate sporozoite quantities. DNA from the pooled, fed mosquitoes was extracted from the whole body of the mosquito according to the protocol from the DNeasy Blood and Tissue kit (Qiagen). The relative level of *Plasmodium* infection was determined by measuring the amount *P. berghei 18s rRNA* compared to mosquito *ribosomal protein S7* (Supplementary Table 1) gene levels in 1 µl of template DNA using the quantitative PCR protocol described above.

For analysis of the hepatic parasite burden, murine livers were harvested 40 h post infection. The whole liver was homogenized in 5 ml of Trizol reagent (Thermo Fisher Scientific). Total RNA was purified from 0.5 ml of the homogenate according to the Trizol protocol and resuspended in 1 ml of molecular biology grade water. Following the extraction of RNA, cDNA was synthesized from 3 μg of total RNA using an iScript kit (Bio-Rad). RT-qPCR was performed in triplicate targeting the *P. berghei 18S rRNA* gene and normalizing gene expression to mouse *beta-actin* (Supplementary Table 1) by the comparative Ct method^62^.

Blood-stage parasitemia was assessed on days 3 through 8 post infection. For this analysis, 10 μl of blood was collected by retro-orbital bleeding and immediately diluted in 500 µl of PBS to prevent clotting. Parasitemia was monitored by measuring the percentage of GFP positive red blood cells on a Stratedigm flow cytometer. Data were analyzed using FlowJo software and compared to the blood of uninfected control mice (v10.2).

### MosGILT activity assay

Recombinant mosGILT activity was assessed by the ability of the enzyme to reduce the disulfide bond of a fluorescent molecule, BODIPY FL L-Cystine (Thermo Fisher Scientific) as previously described^63^. Purified rGILT, human GILT, mouse GILT C2 (CxxS), and lysozyme at 0.05 μM were preactivated with DTT (25 μM) for 10 min at room temperature in a sodium-acetate buffer pH 4.5 or PBS pH 7.4. Serially diluted BODIPY FL L-Cystine substrate (30 μM to 0.234 μM) was added to the enzyme mixture in a 96-well plate (Microfluor 1) and relative fluorescence was monitored over time at 28 °C using a Synergy Mx plate reader (BioTek) with emission/excitation settings of 330/580.

### Two-photon microscopy

Two-photon microscopy was conducted as previously described^34^. Briefly, 5–6-week-old female Swiss Webster mice (Charles River) were lightly anesthetized by intraperitoneal injection of 10% ketamine and 2% xylazine in PBS. Hair from the dorsal ears was removed using a depilatory cream applied for 2 min, then washed gently with PBS. The ear of an individual mouse was gently immobilized over a 14 ml falcon tube covered with double stick tape. Intradermal injection of *P. berghei* (strain NK65) expressing the RedStar fluorescent protein (BEI Resources, MRA-905), was performed under a stereoscope on a 37 °C heated plate. One hundred nanoliters containing 2000 sporozoites/µl preincubated with either rGILT or BSA (80 µg/ml) were injected intradermally into the dorsal ear using glass micropipettes with an 80-µm diameter beveled opening made as described elsewhere^64^ and a Nanoject II Auto-Nanoliter Injector (Drummond). The total number of sporozoites at the injection site ranged between 50 and 200. Two-photon microscopy was performed within the Yale In Vivo Imaging Core Facility using an upright, laser scanning, two-photon microscope operated with a Titanium-Sapphire Laser (Chameleon Vision II, Coherent) tuned to 880 nm and an Olympus ×20 water immersion objective. The mouse was placed on the 37°C heated stage and anesthesia was maintained with an isofluorane-oxygen gas mixture delivered via a nose cone. The injection site images were acquired every 5 s within a 500 µm × 500 µm × 30 µm (*x* × *y* × *z*) field using 10 µm *z*-steps.

Image hyperstacks were compiled using ImageJ software. Imaris software (v. 9.12) was used to semi-automatically track sporozoite movement over time. Imaris was also used to calculate track speed and track straightness. All movies and tracks were reviewed and edited frame by frame to ensure accurate sporozoite tracking.

### Immunoelectron microscopy

Samples were fixed in 4% paraformaldehyde/0.1% gluteraldehyde in phosphate buffered saline for 1 h. They were rinsed in PBS and blocked for 15 min in 50 mM NH_4_Cl + 50 mM glycine and transferred into 2% agar. Cooled samples were trimmed and rinsed in 50 mM maleate buffer pH 5.2 and placed in 2 % uranyl acetate in 50 mM maleate for 1 hour. After rinsing again, they were dehydrated through a graded series of ethanol, 50% to 100%, at 4 °C, then infiltrated in ethanol/HM20 methacrylate resin (EMS), for a few hours followed by several changes of pure 100% HM20 and left overnight on a rotator at 4 °C. Samples were polymerized using UV at 4 °C for 12–18 h. Hardened blocks were sectioned using a Leica UltraCut UC7. Sixty-nanometer-thin sections were collected on formvar/carbon coated grids nickel grids for immuno-labeling.

Grids were placed section side down on drops of 0.1 M ammonium chloride to quench untreated aldehyde groups, then blocked for nonspecific binding on 1% fish skin gelatin in PBS. Single labeled grids were incubated with mouse anti-mosGILT (4C10G9; 1:200 dilution) or an affinity purified anti mouse IgG (H + L) as a control (Jackson ImmunoResearch, 1:200 dilution), both of which required a rabbit anti-mouse bridge (1:200, JacksonImmuno). Fifteen-nanometer Protein A gold (Utrecht Medical Center) was used as a secondary antibody. All grids were rinsed in PBS, fixed using 1% gluteraldehyde for 5 min, rinsed again, and contrast stained using 2% uranyl acetate and lead citrate. Grids were viewed with a FEI Tencai Biotwin TEM at 80 Kv. Images were taken using a Morada CCD and iTEM (Olympus) software.

### Gene silencing

RNA interference of genes expressed in the mosquito SGs was performed as previously described^40^. Double stranded (ds) RNA targeting either a 400 bp region of the *mosGILT* gene or an irrelevant *luciferase* (*luc*) gene from *Renilla reniformis* were transcribed using gene-specific primers designed with a T7 promoter and the MEGAScript RNAi kit (Thermo Fisher Scientific, Ambion; Primers- Supplementary Table 1). *P. berghei*-infected *A. gambiae* 4arr mosquitoes were screened for GFP positive SGs 13 dpi. On 14 dpi, ds*mosGILT* or ds*luc* at 6 mg/ml (1.2 μg total dsRNA) was injected into the thorax of the mosquito using a Nanoject II Auto-Nanoliter Injector (Drummond). Mosquitoes were maintained in paper cups covered with a mesh net at 20 °C and provided 10% sucrose containing 0.05% penicillin/streptomycin for seven days post injection of the dsRNA. SGs were dissected 21 dpi (7 days post dsRNA injection) to monitor the gene expression in the presence of ds*luc* and ds*mosGILT* by both RT-qPCR (Supplementary Table 1) and western blotting as described above. In addition, sporozoites were harvested from the SG tissue of dsRNA injected mosquitoes as described above and used for cell traversal analyses.

### Sporozoite viability assay

*P. berghei* sporozoites were collected from infected mosquitoes as described above and incubated with rGILT (80 µg/ml), BSA (80 µg/ml), or anti-CSP (300 µg/ml) at 4 °C for 1 h before the addition of propidium iodide (Thermo Fisher Scientific, 1 µg/ml) for 10 min. PBS and BSA were used as controls to determine the background level of dead sporozoites under similar incubation conditions. At least 98 sporozoites were counted for each treatment under the EVOS FL Auto Cell Imaging System (Thermo Fisher Scientific). Live sporozoites were identified as GFP positive and propidium iodide negative. The viability of sporozoites was quantified as the percentage of live sporozoites (GFP^+^PI^−^ sporozoites/total sporozoites).

### Primers

All primers utilized in these studies are listed in Supplementary Table 1.

### Statistical analysis

All data analysis, graphing, and statistics were performed in Prism (v7.0, GraphPad Software Inc.).

### Data availability

The mass spectrometry proteomics data files have been deposited to the ProteomeXchange Consortium via the PRIDE^65^ partner repository with the dataset identifier PXD010121. The authors declare that all other data supporting the findings of this study are available within the article and its Supplementary Information files, or are available from the authors upon request.

## Electronic supplementary material

Supplementary Data 2

Supplementary Information

Description of Additional Supplementary Files

Supplementary Data 1
