# Supplementary material for: A mosquito salivary gland protein partially inhibits Plasmodium sporozoite cell traversal and transmission
Source: Nat Commun. 2018 Jul 25;9:2908. doi: 10.1038/s41467-018-05374-3 (PMC6060088; doi:10.1038/s41467-018-05374-3)
Supplement: Supplementary file 2 — Supplementary Information [file 41467_2018_5374_MOESM2_ESM.pdf]

Supplementary Information

A mosquito salivary gland protein partially inhibits *Plasmodium* sporozoite cell traversal and transmission

Schleicher et al.

Supplementary Figures

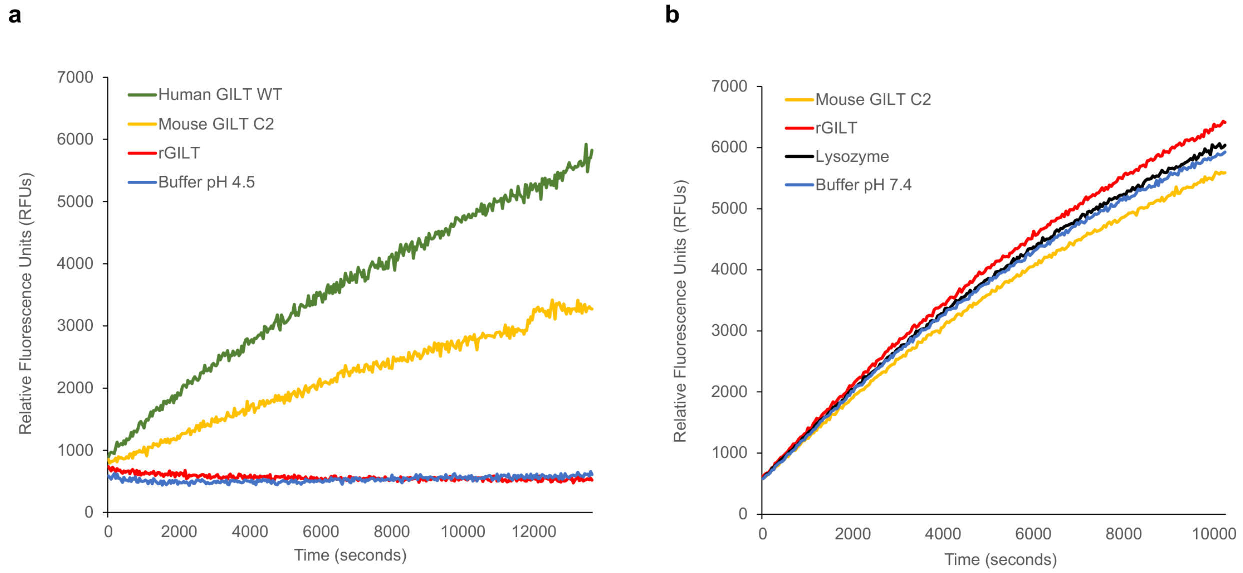

Supplementary Figure 1: Recombinant mosGILT (rGILT) does not have thiol reducing activity. A thiol reduction assay measuring the reduction of fluorescent BODIPY FL L-Cystine over time by rGILT, wild-type human GILT, and mouse GILT with a mutated active site (C2, CxxS) (all 0.05 µM) in either **(a)** a sodium acetate buffer (pH 4.5) or **(b)** a phosphate-buffered saline (PBS, pH 7.4). Graphs represent substrate concentrations of 0.938 µM. For pH 7.4, activity can't be distinguished from the PBS control and is likely caused by the low activity of DTT, a required activating reagent for human GILT. DTT is not active at the acidic pH of 4.5. Lysozyme was used as an additional control at pH 7.4 and has no thiol reducing activity. Data are representative of 2 independent experiments.

a

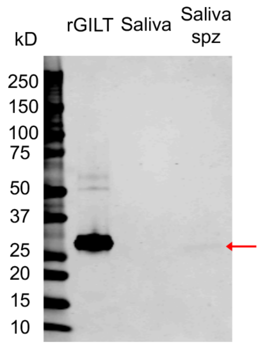

b

Sample	Relative Band Intensity	Estimated amount (ng)
rGILT	59	350
Saliva spz	.69	4.06

# of mosquitoes	mosGILT/ mosquito (ng)	Estimated Volume of saliva (nl)	Concentration in infected saliva (μM)
34	0.12	1	4.5

Supplementary Figure 2: Saliva concentration of mosGILT. (a) Western blot of rGILT, saliva from infected *A. gambiae*, and *P. berghei* sporozoites collected from *A. gambiae* saliva (n=34). Blot was probed with a polyclonal mouse anti-rGILT serum at a 1:1000 dilution. **(b)** The estimated concentration of mosGILT associated with sporozoites in mosquito saliva. The relative intensity of western blot bands for a known amount of rGILT was compared to the intensity of the specific band corresponding to mosGILT associated with sporozoites collected from saliva. Data are representative of 2 independent experiments.

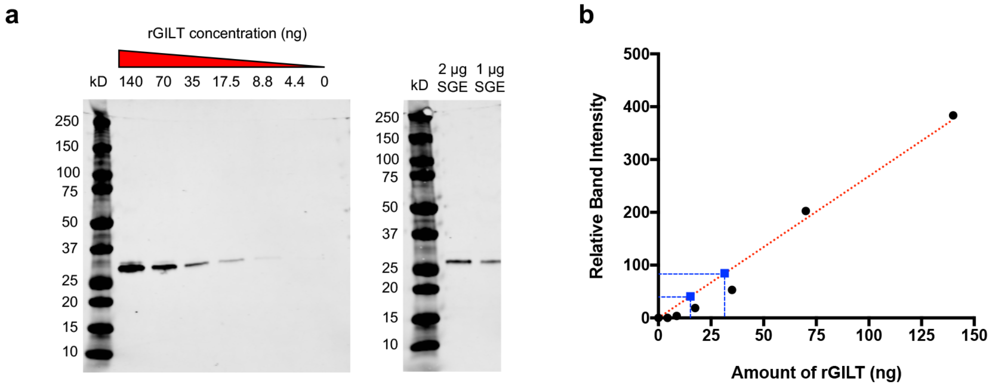

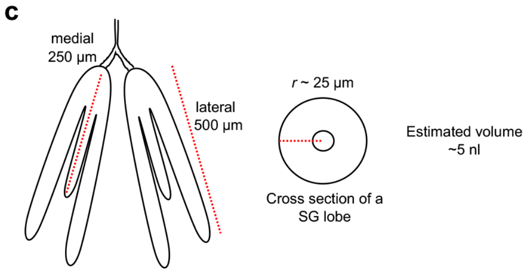

d

Salivary gland extract (SGE, µg)	Relative Band Intensity	Estimated MosGILT (ng)	Concentration (µM)
2	84.6	31.5	235
1	40.8	15.2	112

Supplementary Figure 3: Salivary gland concentration of mosGILT. (a) Western blot of rGILT and *A. gambiae* salivary gland extract (SGE) at known concentrations. Blot was probed with a polyclonal mouse anti-rGILT serum at a 1:1000 dilution. **(b)** Standard curve comparing the relative intensity of western blot bands for known amounts of rGILT and the intensity of the specific bands corresponding to mosGILT from SGE. **(c)** Approximate dimensions of a salivary gland from *Anopheles* mosquitoes. Total volume was estimated by assuming each lobe is similar to a cylinder. **(d)** Calculated intensities from mosGILT SGE bands and predicted endogenous mosGILT concentration of an *A. gambiae* salivary gland. Data are representative of 3 independent experiments.

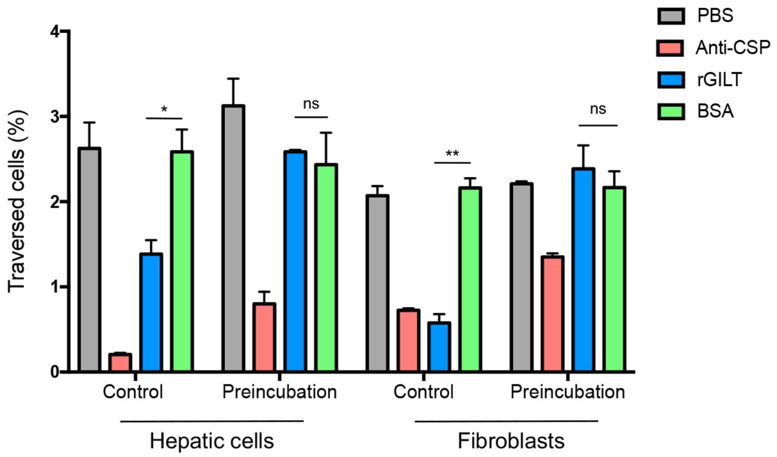

Supplementary Figure 4: Preincubation of host cells in culture with rGILT does not impact sporozoite cell traversal. Sporozoite traversal assay using murine hepatic cells and murine dermal fibroblasts pretreated with rGILT or BSA at 80 µg/ml for 2 hours followed by the addition of untreated sporozoites. *P. berghei* sporozoites were also incubated with rGILT or BSA at 80 µg/ml as controls to compare sporozoite traversal of host cells pretreated with rGILT. Cell traversal by sporozoites was analyzed on a Stratedigm flow cytometer by measuring the percentage of fluorescent dextran positive cells (mean ± SD, unpaired t-test, * $p<0.05$, ** $p<0.005$, ns- not significant). Data are representative of 2 independent experiments.

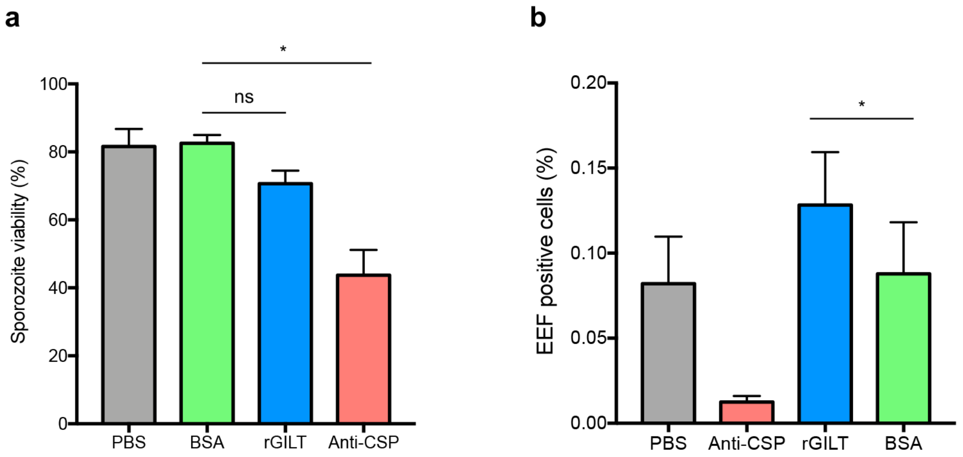

Supplementary Figure 5: Recombinant mosGILT (rGILT) is not toxic to *P. berghei* sporozoites. (a) The viability of *P. berghei* sporozoites was calculated by comparing the percentage of live sporozoites (GFP positive and propidium iodide negative) after incubation with rGILT (80 µg/ml), BSA (80 µg/ml), an anti-circumsporozoite monoclonal antibody (anti-CSP; 300 µg/ml), or a no protein control, PBS (mean ± SD, unpaired t-test, * $p<0.05$, ns- not significant). Data are representative of 2 independent experiments. **(b)** *P. berghei* sporozoites were incubated with rGILT (80 µg/ml) and then combined with murine hepatic cells for 48 hours. The percentage of EEF-positive cells (positive for GFP signal) was measured by flow cytometry. BSA (80 µg/ml) and anti-CSP (300 µg/ml) were used as controls (mean ± SD, unpaired t-test, * $p<0.05$). Data are representative of more than 3 independent experiments.

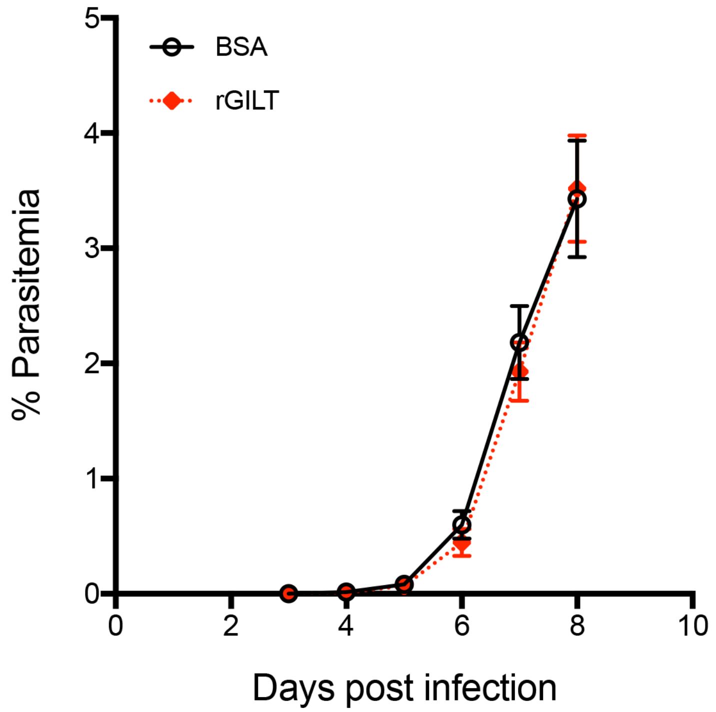

Supplementary Figure 6: Blood-stage parasitemia is not altered after infection with rGILT-treated sporozoites. After the intradermal injection of 2,000 *P. berghei* sporozoites incubated with either rGILT or BSA (100 µg/ml each), the percentage of blood-stage *Plasmodium* was monitored 3-8 days post infection (dpi) with flow cytometry by the detection of GFP positive infected red blood cells. Data were pooled from two independent experiments (mean ± SD, n = 17 BSA, n = 17 rGILT, ns- not significant).

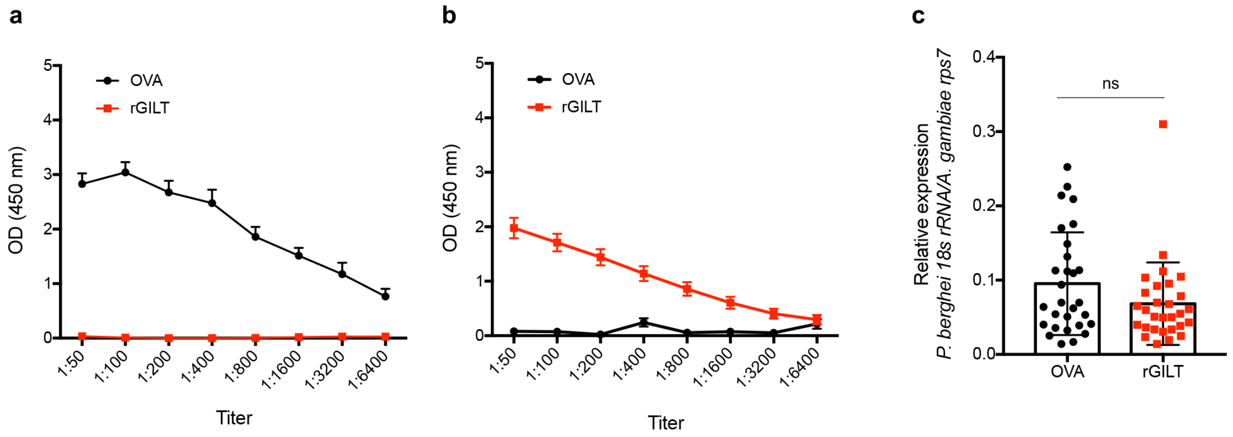

Supplementary Figure 7: Analysis of mice and mosquitoes used during active immunization studies. Sera from individual mice immunized with either OVA or rGILT were incubated on 96-well microtiter plates coated with 3 µg/ml of **(a)** OVA or **(b)** rGILT (n=10 mice/group; one representative experiment from three total experiments). Titers were compared by measuring the absorbance of each well spectrophotometrically at an OD of 450 nm (mean ± SEM). **(c)** Each point represents the mosquitoes (two or three) which fed on actively immunized mice. *Plasmodium* DNA levels from the whole body of each mosquito were assessed by measuring the *P. berghei 18S rRNA* gene and normalizing to the *A. gambiae ribosomal protein S7* gene using RT-qPCR. (OVA n = 29, rGILT n=29). Data are pooled from three independent experiments (mean ± SD, unpaired t-test, ns- not significant).

Supplementary Tables

Supplementary Table 1: Primer Information

Primer Name	Sequence 5'-3'	Length	Product Size (bp)
AgActinF	GAAGGCTAACCGCGAGAAGATG	22	134
AgActinR	CGCCGGAGTCCAGCACGATA	20	134
AgRPS7F	GCGCCGCATTCTGCCCAAAC	20	148
AgRPS7R	GACGCGGATACGCTTGCCGA	20	148
Pb18sF	CCGACTAAGTGTTGGATGAAAA	22	119
Pb18sR	TACTCGCCCCAGAACCCAAAGA	22	119
MactinF	ACGGCCAGGTCATCACTATTG	21	131
MactinR	ACTATGGCCTCAGGAGTTTTGTCA	24	131
4551F	ATGCTGTTCAAAAGCTTGCTTCT	23	762
4551R	AGTAAGATTCAATACCCCCACTGG	24	762
4551F1	TGGCGAAGGAGCTCAAAAAG	20	123
4551R1	TGCCGTAACACTCGTTTTCG	20	123
GILT_NcoI	AAAAAACCATGGCAATCAAAAGTGCCCGTATACGT	35	669
GILT_BamHI	AAAAAAGGATCCCAATCAAAAGTGCCCGTATACGT	35	669
GILT_NheI	AAAAAAGCTAGCCAATCAAAAGTGCCCGTATACGT	35	669
GILT_NotI	AAAAAAGCGGCCGCGAGCAGTCTAGAGATGAGAACAGC	38	669
GILTPMTNotI	AAAAAAGCGGCCGCAAGAGCAGTCTAGAGATGAGAACAGC	40	669
GiltC32SF	TACTACGAATCGCTCTCCCCGGATAGTGCTCGG	33	-
GiltC32SR	CCGAGCACTATCCGGGGAGAGCGATTCGTAGTA	33	-
GILTdCF_NcoI	AAAAAACCATGGCAATCAAAAGTGCCCGTATACGT	35	588
GILTdCR_NotI	AAAAAAGCGGCCGCAAGAGACACTCGACGGGCTGT	35	588
dslucF	TAATACGACTCACTATAGGGAGAAACGGATGATAACTGGTCCGC	44	412
dslucR	TAATACGACTCACTATAGGGAGAACATCTACTACACTTTCAGCG	44	412
dsmosGILTF	TAATACGACTCACTATAGGGAGAGGGTAACTCGTATCAGCCGA	43	412
dsmosGILTR	TAATACGACTCACTATAGGGAGAAACAGCGACCACAGTAACGA	43	412
