# Supplementary material for: A mosquito salivary gland protein partially inhibits Plasmodium sporozoite cell traversal and transmission
Source: Nat Commun. 2018 Jul 25;9:2908. doi: 10.1038/s41467-018-05374-3 (PMC6060088; doi:10.1038/s41467-018-05374-3)
Supplement: Supplementary file 3 — Description of Additional Supplementary Files [file 41467_2018_5374_MOESM3_ESM.pdf]

Description of Additional Supplementary Files

File Name: **Supplementary Data 1**
Description: **Protein identifications from sporozoites collected from mosquito saliva.**
Sporozoites (*Plasmodium berghei and Plasmodium falciparum*) collected from *Anopheles gambiae* or *Anopheles stephensi* were analyzed by MS/MS and compared to naïve saliva samples prepared in the same way. Mosquito proteins identified with sporozoites were compared between the two groups over three independent replicates. This analysis was used to determine whether there are any unique vector proteins interacting with sporozoites in saliva that are not normally present in naïve saliva.

File Name: **Supplementary Data 2**
Description: **Protein identifications from *Anopheles gambiae* salivary glands.**
The total protein content of salivary glands dissected from blood fed *A. gambiae* were analyzed by MS/MS. Total protein identifications were ranked by spectral abundance and compared across three biological replicates using Scaffold (v. 4.8.4, Proteome Software Inc.).
